# Inflammatory Blood Biomarker Kynurenine Is Linked With Elevated Neuroinflammation and Neurodegeneration in Older Adults: Evidence From Two 1H-MRS Post-Processing Analysis Methods

**DOI:** 10.3389/fpsyt.2022.859772

**Published:** 2022-04-11

**Authors:** Wouter A. J. Vints, Simona Kušleikiene, Samrat Sheoran, Milda Šarkinaite, Kristina Valatkevičiene, Rymante Gleizniene, Mindaugas Kvedaras, Kazimieras Pukenas, Uwe Himmelreich, Vida J. Cesnaitiene, Oron Levin, Jeanine Verbunt, Nerijus Masiulis

**Affiliations:** ^1^Department of Health Promotion and Rehabilitation, Lithuanian Sports University, Kaunas, Lithuania; ^2^Department of Rehabilitation Medicine Research School Caphri, Maastricht University, Maastricht, Netherlands; ^3^Centre of Expertise in Rehabilitation and Audiology, Adelante Zorggroep, Hoensbroek, Netherlands; ^4^Department of Radiology, Medical Academy, Lithuanian University of Health Sciences, Kaunas, Lithuania; ^5^Biomedical MRI Unit, Department of Imaging and Pathology, Group Biomedical Sciences, Catholic University Leuven, Leuven, Belgium; ^6^Movement Control & Neuroplasticity Research Group, Group Biomedical Sciences, Catholic University Leuven, Heverlee, Belgium; ^7^Department of Rehabilitation, Physical and Sports Medicine, Institute of Health Science, Vilnius University, Vilnius, Lithuania

**Keywords:** aging, cognition, obesity, gray matter volume, neurochemicals, cerebral metabolite ratios, inflammation, brain imaging

## Abstract

**Rationale and Objectives:**

Pro-inflammatory processes have been argued to play a role in conditions associated with cognitive decline and neurodegeneration, like aging and obesity. Only a limited number of studies have tried to measure both peripheral and central biomarkers of inflammation and examined their interrelationship. The primary aim of this study was to examine the hypothesis that chronic peripheral inflammation would be associated with neurometabolic changes that indicate neuroinflammation (the combined elevation of myoinositol and choline), brain gray matter volume decrease, and lower cognitive functioning in older adults.

**Materials and Methods:**

Seventy-four older adults underwent bio-impedance body composition analysis, cognitive testing with the Montreal Cognitive Assessment (MoCA), blood serum analysis of inflammatory markers interleukin-6 (IL-6) and kynurenine, magnetic resonance imaging (MRI), and proton magnetic resonance spectroscopy (^1^H-MRS) of the brain. Neurometabolic findings from both Tarquin and LCModel ^1^H-MRS post-processing software packages were compared. The regions of interest for MRI and ^1^H-MRS measurements were dorsal posterior cingulate cortex (DPCC), left hippocampal cortex (HPC), left medial temporal cortex (MTC), left primary sensorimotor cortex (SM1), and right dorsolateral prefrontal cortex (DLPFC).

**Results:**

Elevated serum kynurenine levels were associated with signs of neuroinflammation, specifically in the DPCC, left SM1 and right DLPFC, and signs of neurodegeneration, specifically in the left HPC, left MTC and left SM1, after adjusting for age, sex and fat percentage (fat%). Elevated serum IL-6 levels were associated with increased Glx levels in left HPC, left MTC, and right DLPFC, after processing the ^1^H-MRS data with Tarquin. Overall, the agreement between Tarquin and LCModel results was moderate-to-strong for tNAA, tCho, mIns, and tCr, but weak to very weak for Glx. Peripheral inflammatory markers (IL-6 and kynurenine) were not associated with older age, higher fat%, decreased brain gray matter volume loss or decreased cognitive functioning within a cohort of older adults.

**Conclusion:**

Our results suggest that serum kynurenine may be used as a peripheral inflammatory marker that is associated with neuroinflammation and neurodegeneration, although not linked to cognition. Future studies should consider longitudinal analysis to assess the causal inferences between chronic peripheral and neuroinflammation, brain structural and neurometabolic changes, and cognitive decline in aging.

## Introduction

Looking back at how the world's population has changed in the last four decades, two major trends have been noticed; (a) the increase in the proportion of older adults and (b) the increase in the prevalence of overweight and obesity. Indeed, first of all, the subpopulation of older adults is rapidly increasing worldwide and is prospected to continue to increase (1). With it, the increasing amount of care-needing elderly places high demands on our health care services (1). At the same time, there is a shift in the prevalence of diseases in the group of older adults showing an increase in chronic care-demanding illnesses. A comparison of diseases in older adults in 2016 showed that dementias displayed the largest increase as a cause of disability and second largest increase as a cause of mortality in the last 16 years (2). Importantly, Mayeux and Stern (3) hypothesized that the burden of dementia, with Alzheimer's disease as the leading cause of dementia, will double every 20 years until at least 2040. Secondly, a dramatic increase in the prevalence of overweight and obesity has taken place in the last four decades. At this rate, the majority of the world's adult population will be overweight or obese by 2030 (4). Of interest, not only older age, but also obesity has been associated with cognitive decline, as was reported after adjusting for age and educational level (5). Moreover, obese individuals were found to have an increased risk of Alzheimer's disease (6). Furthermore, life-style interventions targeted to reduce obesity, like regular physical exercise and a healthy diet, are also suggested to be promising strategies to prevent cognitive decline and dementia (7).

A common mechanism that has been suggested to be a major player in age- and obesity-related cognitive decline is a chronic state of inflammation (8–10). Studies have measured increased levels of inflammatory markers and activated immune cells in blood samples and tissue biopsies in old and obese individuals (11, 12). In the context of aging, ‘inflammaging' refers to the gradual transition into a chronic pro-inflammatory stage that is reported when people get older (11). It is thought that inflammaging arises at least partly from the release of inflammatory markers by senescent cells, which accumulate over time (13). The senescence program makes old cells stop dividing to prevent malignant transformation (14), but also alters secretory activity so that they start releasing a range of inflammatory markers and attract and activate immune cells (12). In the context of obesity, chronic inflammation is suggested to arise from adipose cells. Especially from visceral fat, which is considered one of the largest contributors to the release of inflammatory markers in the human body (15, 16).

Of importance, these signs of elevated inflammation can also be found in the brain, both with aging (17) and obesity (18). Within the central nervous system, glial cells, which include microglia and astrocytes, are the main immune cells involved in local neuroinflammation (17). Stereological findings have shown an age-related increase in the number of glial cells in the frontal and temporal cortex (19). Moreover, studies have shown that microglia change to a more proinflammatory phenotype with age (20, 21). In the context of obesity, neuroinflammation in the hypothalamus is consistently reported (9), but also other brain regions are affected, such as the hippocampus, amygdala, cerebral cortex and cerebellum, as has been shown in rodent studies (10, 22–24). In the areas with increased neuroinflammation, activated microglia release inflammatory markers to attract more inflammatory cells and initiate repair mechanisms, further increasing inflammation in the brain (25–28). This may cause damage to the surrounding neurons and may impair synaptic signaling, which eventually results in cognitive impairment (8, 17, 29).

It is considered that peripheral inflammation and neuroinflammation are closely linked, even though the brain and the rest of the body are separated from each other by the blood–brain barrier. Indeed, peripheral and central inflammatory signals were previously found to interact via blood and neural routes of communication (30–37). For example, studies have shown that a peripheral inflammatory challenge activated microglial cells and drastically altered neural activity (35, 38). After blocking peripheral cytokine production, neuroinflammation was also altered and this was found to result in cognitive improvements (39, 40). Inversely, traumatic brain injury was reported to induce an inflammatory response in the liver, causing liver damage (41–43). Recently, the neurobiological link between peripheral inflammation, neuroinflammation, and cognitive aging was tested for the first time by Lind et al. (44). Firstly, in a previous study, they used proton magnetic resonance spectroscopy (^1^H-MRS) to search for signs of neuroinflammation and discovered a link between cognitive aging and elevated levels of the glia-related neurometabolites, total choline (tCho), myoinositol (mIns), and total creatine (tCr) in the anterior cingulate cortex, hippocampus, and thalamus. Secondly, Lind et al. (45) correlated these findings to blood levels of the pro-inflammatory markers C-reactive protein (CRP), interleukin-8 (IL)-8, and tumor necrosis factor-α (TNF-α) in young, middle-aged, and older adults. They reported that circulating levels of CRP and IL-8 were elevated in older adults. The CRP was positively correlated to thalamic mIns levels and IL-8 was positively correlated with tCho in the anterior cingulate cortex as well as with mIns in the hippocampus. Moreover, they observed a negative correlation between CRP levels and visuo–spatial working memory performance.

In addition to CRP, IL-8, and TNF-α, other markers of inflammation have previously been linked to aging, obesity, and cognitive impairment. For example, IL-6 is extensively studied as a marker of inflammaging and has even been called the “gerontologist's cytokine” (46). The levels of IL-6 are increased in older and in obese adults (47). A meta-analysis including seven longitudinal studies presented evidence that subjects with high circulating IL-6 levels were 1.42 times more likely to experience global cognitive decline after a 2–7 year follow-up (48). However, IL-6 seems also to be involved in anti-inflammatory actions and is therefore suggested to be an important regulator of the inflammatory response (49). Another very interesting inflammatory marker is kynurenine, as the enzyme indolamine 2,3-dioxygenase, which converts tryptophan into kynurenine in the liver, is upregulated in a state of high inflammation (50). Increased indolamine 2,3-dioxygenase activity and elevated levels of kynurenine were associated with higher levels of CRP, IL-1β, IL-6, IL-8, TNF-α, and Interferon-gamma (IFN-γ), while the anti-inflammatory cytokines IL-4 and IL-10 counteracted the increase in kynurenine levels (51–54). Through this pathway, inflammation results in a reduction in tryptophan, a precursor of serotonin and may cause sickness behavior and depression (55). Furthermore, kynurenine metabolites have been shown to induce dysregulation of glutamatergic pathways, leading to excitotoxic neural damage (56). The higher levels of kynurenine were associated with obesity (57), with cognitive decline in older adults (58) and with striatal and hippocampal volume loss in psychiatric disorders (59). In a model applying Alzheimer mouse, peripheral inhibition of the kynurenine pathway prevented neurodegeneration and memory deficits (60).

The primary aim of this study was to investigate the association between peripheral inflammatory markers measured from serum samples and signs of neuroinflammation measured with ^1^H-MRS in a cohort of older adults and secondarily to relate peripheral inflammatory markers to brain gray matter volume (GMV) and cognitive function, as measured with the Montreal Cognitive Assessment (MoCA). The MoCA is considered a sensitive screening tool to evaluate the risk of mild cognitive impairment in the geriatric population (61, 62). We built our analysis starting first with assessing the relationship between the demographic characteristics of the subjects included in our study and cognitive function. Based on the existing literature, we also expect to find that older age and higher body fat percentage (fat%) are associated with cognitive deficit (5, 8). Of importance, studies have also shown that cognitive performance depends on the subject's educational level; for example, see Malek-Ahmadi et al. (63), and fat% greatly differs between sexes. Also in older adults, fat% is generally larger in women than in men (64). Secondly, we will correlate age, fat%, and cognition to peripheral inflammatory markers, brain GMV, and neurometabolic changes. Here, we hypothesize that older age, higher fat% and lower cognitive functioning are associated with higher levels of peripheral inflammation, a loss of brain GMV, a decrease in markers of neural integrity and an increase in markers of neuroinflammation. Thirdly, we test the hypothesis that higher levels of peripheral inflammation are associated with brain volume loss, and eventually our study's primary endpoint that peripheral inflammation is associated with neurometabolic changes indicating brain inflammation. This investigation elaborates the findings of Lind et al. (45), since we used different peripheral serum biomarkers, added brain GMV measurements, examined more brain regions with ^1^H-MRS, and used the MoCA as a more general screening instrument for mild cognitive impairment in older adults. Furthermore, we took into account the effect of age, fat%, and sex and excluded subjects with neurological and psychiatric disorders. Finally, quantification of neurometabolites such as mIns and Glx may not be consistent across vendors and algorithms (65). Therefore, we used LCModel and Tarquin, two linear-combination modeling algorithms commonly implemented for the quantification of ^1^H-MRS spectra, to examine the robustness of our neurometabolic estimates and the predictability of their associations with the other biomarkers. Ultimately, this paper may lead to a better understanding of the expected detrimental effect of inflammation on brain health and cognitive function.

## Materials and Methods

### Participants and Setting

A total of 74 apparently healthy adults aged 60 years and older were recruited in Kaunas, Lithuania. The recruitment strategies included presentations in local community organizations, contacting subjects from a list of patients provided by general practitioners and volunteers from previous studies. Candidates were invited for an interview in the primary care center, Saules Family Medical Center, before inclusion. The exclusion criteria were a diagnosis of a neurological disorder, including stroke, epilepsy, multiple sclerosis, traumatic brain injury, brain tumor or neurodegenerative diseases like dementia. Furthermore, we excluded subjects with alcohol or drug abuse, diabetes, psychiatric disorders such as depression, or usage of psychopharmacological drugs in the last 5 years and oncologic disorders or a history of chemotherapy use. Finally, we applied the exclusion criteria for magnetic resonance imaging (MRI) studies as formulated by the checklist of the Department of Radiology, Lithuanian University of Health Science, including metal or MR-incompatible implants and claustrophobia. The participants indicated that they did not participate in any regular exercise program in the last 6 months, but were able to perform 10 sit-ups. The participants could voluntarily withdraw from the study at any time.

### Demographic and Clinical Characteristics

All participants were asked to report age, sex, smoking status, and educational level (i.e., basic education, secondary education or higher education). Furthermore, the MoCA test was conducted by a qualified mental health care specialist (co-author SK). The MoCA test is a brief cognitive screening instrument developed by Nasreddine et al. (61). It contains 12 items measuring seven cognitive domains as follows: Executive functioning; visuospatial abilities; language; attention, concentration and working memory; abstract reasoning; memory and orientation. All items add up to a total score with a maximum of 30 points, where a higher score indicates better cognitive functioning. This screening tool is widely used and is especially considered as a reliable and sensitive tool to evaluate the risk of mild cognitive impairment in the geriatric population (61, 62). At last, we assessed the subjects' body mass index (BMI) and measured their body fat% using leg-to-leg bio-impedance analysis (Tanita TBF-300-A).

### Blood Analysis

Venous blood samples were drawn by a qualified medical professional at the antecubital vein. All blood samples were collected between 9 a.m. and 1 p.m. in 5 ml serum separation gel tubes. After blood collection, the tubes were gently inverted 8–10 times and kept at room temperature for 30 min until centrifugation for 15 min at 4,000 *g* centrifugal force. After centrifugation, serum was aliquoted into 1.5 ml polypropylene tubes and immediately frozen and stored at −80°C in the refrigerator compartment of the laboratory of the Lithuanian Sports University Institute of Sports Science and Innovation until further analysis. Enzyme-linked immunosorbent assay (ELISA) tests for the assessment of the circulating levels of IL-6 and kynurenine were analyzed with spectrophotometry (Spark 10M, Tecan Group Ltd., Zürich, Switzerland) by an experienced researcher (co-author MK).

*IL-6 measurement*. The IL-6 concentrations were measured using a commercially available ELISA kit purchased from DIAsource ImmunoAssays S.A., Belgium (KAP1216). It was a sandwich immunometric assay utilizing recombinant human cytokines and antibodies raised against recombinant human cytokines. The lower limit of the detection being 2 pg/ml. Absorbance was measured using a spectrophotometer at 450 nm absorbance.

*Kynurenine measurement*. Kynurenine concentrations were measured using a commercially available ELISA kit purchased from MyBiosource, Inc., USA. Lower limit of detection being 45.7 ng/ml.

### Brain Imaging and ^1^H-MRS

Brain scanning consisted of whole brain MRI and ^1^H-MRS with voxels in five brain regions with a total length of about 90 min per subject. An MRI was performed using a Siemens 3T Skyra scanner (Siemens Healthineers, Erlangen, Germany) with a 32-channel receiver head coil. A high-resolution T1-weighted (T1W) structural MR image [(repetition time (TR) = 2,200 ms, echo time (TE) = 2.48 ms, 0.9 × 0.9 × 1.0 mm3 voxels, field of view: 230 × 256 mm, number of sagittal slices = 176)] was used to acquire a 3D magnetization prepared gradient echo (MPRAGE). A T2-weighted (T2W) turbo-spin echo scan, a fluid-attenuated inversion recovery (FLAIR) and a susceptibility weighted imaging (SWI) were reviewed for brain lesions by an experienced radiologist with more than 10 years of experience (co-author KV).

Total GMV and GMVs of dorsal posterior cingulate cortex (DPCC), left and right hippocampal cortex (HPC), left middle temporal cortex (MTC), left primary sensorimotor cortex (SM1) and right dorsolateral prefrontal cortex (DLPFC) were analyzed using FreeSurfer v7.1.1 (Harvard, MA, USA, http://surfer.nmr.mgh.harvard.edu/) on isotropic 3D T1W images of 0.9 mm slice thickness. The DPCC GMV was calculated by combining the Freesurfer region volumes right and left posterior cingulate. For the left and right HPC GMV, the Freesurfer regions left and right whole hippocampus were used. The left MTC GMV was calculated from middle temporal Freesurfer site of the left hemisphere. Left SM1 GMV was the combined volume of Freesurfer precentral area of frontal lobe and postcentral area of parietal lobe of the left hemisphere. Finally, the right DLPFC GMV was calculated by combining the Freesurfer region volumes of left rostral and caudal middle frontal regions.

^1^H-MRS spectra were acquired in five voxels using a Point RESolved Spectroscopy (PRESS) sequence (TR = 2,000 ms, TE = 30 ms, number of averages = 128, spectral bandwidth = 2,000 Hz, and data size = 1,024 points) with excitation water suppression (sequence svs_se_30). The unsuppressed water signal was also acquired to measure absolute metabolite concentrations using the same acquisition parameters. The regions of interest where voxels were placed included DPCC, left HPC, left MTC, left SM1, and right DLPFC and corresponded to the regions used to calculate GMVs. The DPCC voxel was placed in the midline of both posterior parts of cingulate cortex in axial plane anterior to precuneus and parieto-occipital sulcus, corresponding to the Freesurfer sites used to calculate DPCC GMV. The hippocampal voxel was centered over the whole hippocampus in left hemisphere in the medial part of the temporal lobe anterior to lateral ventricle, corresponding to the Freesurfer region used to calculate left HPC GMV. The MTC voxel was placed in the left middle part of temporal lobe over the middle temporal gyrus caudal to superior temporal gyrus and cranial to inferior temporal gyrus, corresponding to the Freesurfer site used to measure left MTC GMV. The SM1 voxel was centered over the left hand-knob, corresponding to the Freesurfer sites used to calculate left SM1 GMV. The DLPFC voxel was placed over the right middle frontal gyrus inferior to the superior frontal sulcus and anterior to the precentral sulcus, corresponding to the Freesurfer sites used to calculate right DLPFC GMV. The voxel sizes were: (i) 1.6 × 1.6 × 1.6 cm3 in the DPCC, the left SM1, and the right DLPFC voxels, (ii) 20 × 12 × 16 cm^3^ in the left MTC and (iii) 26 × 12 × 12 cm^3^ in the left HPC. The MR spectra were processed using the totally automatic robust quantification in nuclear MR (TARQUIN, version 4.3.10) and the linear combination of model spectra (LCModel, version 6.3.1-R). These two post-processing software packages are widely used (65). However, LCModel may offer a more accurate quantification of Glx and macromolecular constituents (66). We presented Tarquin results after lipid signal extraction using the lipid-filtering preprocessing option prior to quantification, as recommended by Near et al. (67). However, LCModel has no lipid filter option at the preprocessing phase. Therefore, we present right DLPFC Tarquin results with no filtering of lipids compared to LCModel results in Supplementary Table 1. This table confirms that the same conclusion can be drawn as when lipids were filtered before processing with Tarquin, as shown in Table 1, see results section. Only spectra with linewidths <12 Hz and signal-to-noise ratio >5 were included in the statistical analyses, other values were considered missing. For spectra that were processed with LCModel, all included neurometabolites were quantified with a Cramér-Rao lower bound <20%. ^1^H-MRS spectra were visually checked to ensure the absence of artifacts prior to quantification. Quantifiable neurometabolites were (1) total NAA (tNAA) composed of *N*-acetyl aspartate and *N*-acetyl glutamate, (2) tCr composed of creatine and phosphocreatine, (3) tCho composed of phosphorylcholine and glycerophosphocholine, (4) mIns, and (5) the glutamate–glutamine complex (Glx).

**Table 1:** Agreement between LCModel and Tarquin ^1^H-MRS post-processing software packages.

		**tNAA**	**tCho**	**Glx**	**mIns**	**tCr**
DPCC	*R*-value ICC	0.699*** 0.666***	0.597*** 0.514***	0.097 0.030	0.687*** 0.466***	0.789*** 0.408***
Left HPC	*R*-value ICC	0.574*** 0.354***	0.619*** 0.516***	0.228 0.074	0.358** 0.314**	0.641*** 0.606***
Left MTC	*R*-value ICC	0.515*** 0.407***	0.692*** 0.655***	−0.011 −0.003	0.726*** 0.474***	0.485*** 0.411***
Left SM1	*R*-value ICC	0.579*** 0.541***	0.790*** 0.661	0.376** 0.161**	0.817*** 0.663***	0.742*** 0.213***
Right DLPFC	*R*-value ICC	0.486*** 0.472***	0.852*** 0.795***	0.187 0.061	0.751*** 0.559***	0.678*** 0.227***

*Pearson R and ICC values are presented for the correlation between Tarquin and LCModel measurements*.

*p < 0.05, **p < 0.01, ***p < 0.001; DLPFC, dorsolateral prefrontal cortex; DPCC, dorsal posterior cingulate cortex; Glx, glutamate–glutamine complex; HPC, hippocampal cortex; ICC, intraclass correlation coefficient; mIns, myoinositol; MTC, medial temporal cortex; SM1, primary sensorimotor cortex; tCho, total choline; tCr, total creatine, tNAA, total N-acetyl aspartate.

Water-referenced levels of tNAA, tCr, tCho, mIns, and Glx were quantified for each voxel location and ratios relative to tCr were calculated. For tNAA also, the ratio relative to mIns was calculated. Neuroinflammation and gliosis were reported in association with elevated levels of mIns, tCr, and tCho (44, 68, 69). The concentrations of these neurometabolites are significantly higher in glial cells than in neurons and the concomitant increase in mIns and tCho is considered to indicate glial proliferation (70). In addition, decreased concentrations of tNAA are considered a robust marker of neurodegeneration (71). The ratio of tNAA relative to mIns is an interesting marker for neurodegenerative processes that involve inflammation, as it indicates a combined loss of neural integrity (tNAA) and increase in neuroinflammation (mIns) (72).

### Statistical Analysis

Statistical analysis was performed using IBM SPSS Statistics version 27 (SPSS Inc, Chicago USA). We used parametric tests where possible. In case this was not appropriate due to not meeting normality assumptions, data was log transformed, as was the case for IL-6 measurements. After data inspection and cleaning bad quality measurements (see criteria in 4. Brain imaging and ^1^H-MRS), the agreement between LCModel and Tarquin ^1^H-MRS post-processing software packages was evaluated using intraclass correlation coefficients (ICC, single measure two-way random effects model for absolute agreement). Subject demographics were correlated to MoCA test scores using linear regression analysis for continuous markers and two-sided independent *t*-tests to compare MoCA test results between two groups. Linear regression was used to correlate age with levels of serum inflammatory markers, brain GMV and neurometabolite levels. Multiple linear regression analysis was used to investigate the association between MoCA score, adjusted for age and educational level, or fat%, adjusted for age and sex and peripheral inflammatory marker levels, brain GMV, and neurometabolite levels. Finally, multiple regression analysis was used to investigate the association between peripheral inflammatory marker levels, adjusted for age, sex, and fat% and brain GMV and neurometabolite levels. For the purpose of this exploratory study, *p*-values below 0.05 were considered statistically significant. In addition, we presented which of these *p*-values survived correction for multiple testing with false discovery rate (FDR) analysis (Benjamini and Hochberg adjustment) (73). In the FDR procedure, every *p*-value is compared against a sequentially weighted threshold on all *p*-values. The FRD procedure was done multiple times for each of the independent variables age, MoCA, fat%, IL-6, IL-6 after exclusion of an outlier, and kynurenine; and separately for LCModel results, Tarquin results, and the combination of all other dependent variables for all *p*-values presented in Supplementary Tables 3–10. It should be noted that the results surviving the multiple testing adjustment are strong, whereas interpretation of the remaining results should be made with caution.

## Results

### Magnetic Resonance Data Quality and Analysis

**Figure 1:**
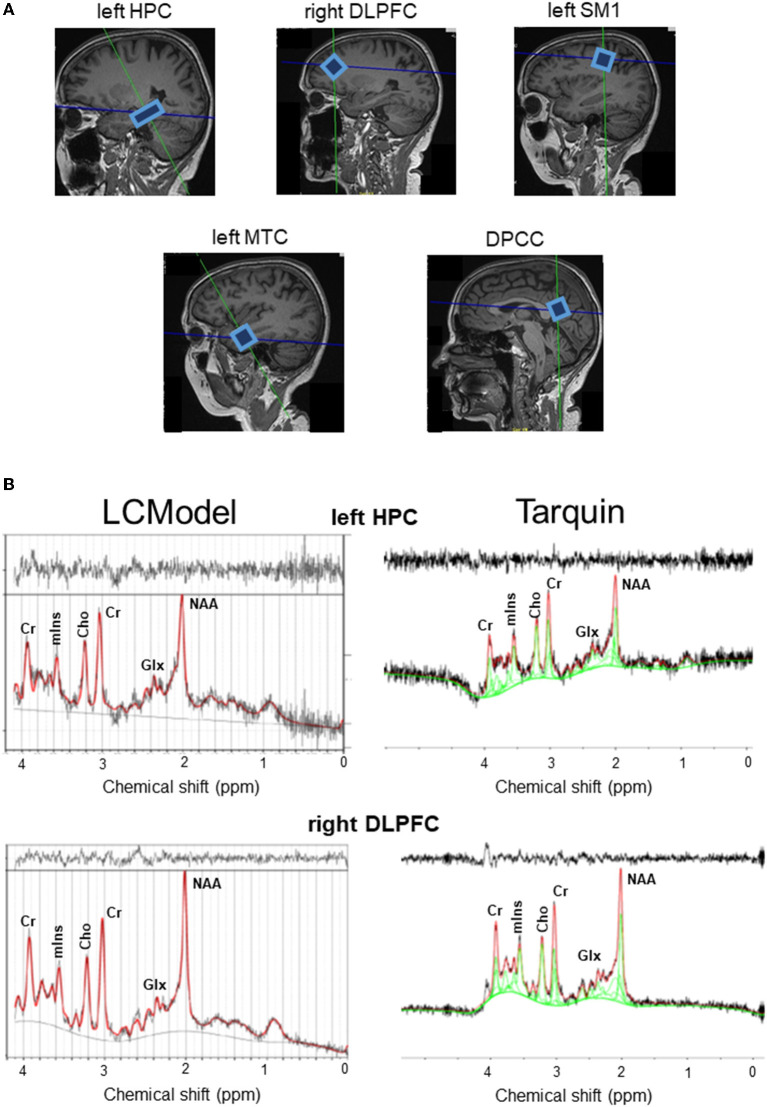
**(A)** Example voxel positions and spectra from a representative subject. **(B)** Raw (black curve) and fitted (red curve) spectra from LCModel (left hand side panel) and Tarquin (right hand side panel) are illustrated for the left HPC and right DLPFC. Cho, total choline; Cr, creatine + phosphocreatine; DLPFC, dorsolateral prefrontal cortex; DPCC, dorsal posterior cingulate cortex; Glx, glutamate–glutamine complex; HPC, hippocampal cortex; mIns, myoinositol; MTC, medial temporal cortex; NAA, *N*-acetyl aspartate; SM1, primary sensorimotor cortex.

Of all included subjects, 68 (91.9%) underwent complete brain scanning sessions with MRI and ^1^H-MRS. The reasons for missing data were inclusion in a pilot ^1^H-MRS scanning session using stimulated echo acquisition mode (STEAM) sequence (*n* = 2) or scanning cessation due to claustrophobia, unrest, discomfort, or pain during scanning (*n* = 4). After excluding data of bad quality, a complete dataset of all MRI and MRS values was attained for 33 subjects. Further analysis was done each time with all the data available, which differed depending on the measurement. To know specifically from how many subjects the data was used for each of the associations assessed below, Supplementary Table 2 lists the number of subjects with good quality MRI and ^1^H-MRS values, with ^1^H-MRS values presented per region and compared between LCModel and Tarquin (see Figure 1).

Overall, the agreement between tNAA, tCho, mIns, and tCr measurements postprocessed with LCModel compared to measurements after processing with Tarquin were strong (*R* or ICC > 0.750) or moderate (0.500 < *R* or ICC < 0.750), with exception of weak correlations (0.250 < *R* < 0.500) for tNAA levels in the right DLPFC, mIns levels in the left HPC and tCr levels in the left MTC; or low ICC values (0.250 < ICC < 0.500) for tNAA levels in the left HPC, left MTC and right DLPFC, mIns levels in the DPCC, left HPC, left MTC and tCr levels in the DPCC, left MTC, left SM1 and right DLPFC. The agreement of Glx levels between the two models was very weak (*R* or ICC < 0.250) in all regions of interest, except for a weak correlation in the left SM1 (*R* = 0.376); for details, see Table 1.

### Subject Characteristics

Subject characteristics are described in Table 2. The mean (SD) MoCA score was 25.2 (2.8), ranging from 19 to 30. The age of the included subjects ranged from 60 to 85 years old. Older aged adults scored significantly lower on the MoCA test (*R*^2^ = 0.175, *p* < 0.001). Fat% ranged from 3.4–51.6% and BMI from 19.2–47.9. As expected, fat% was highly dependent on sex (*p* < 0.001), with mean (SD) fat% in men 26.3% (8.7) and in women 35.8% (7.8). The BMI did not significantly differ between sexes (*p* = 0.361), with mean (SD) BMI in men 28.6 (8.7) and in women 27.5 (4.5). Fat% and BMI, adjusted for age and sex, were not significantly correlated with MoCA scores (*p* = 0.202, *p* = 0.127, respectively). Older age was significantly correlated with lower fat% (*R*^2^ = 0.143, *p* = 0.001), but not with BMI (*R*^2^ = 0.019, *p* = 0.128).

**Table 2:** Subject characteristics and their relation to MoCA score.

	**Total** **(*n* = 74)**	**β**	**Regression** ***p*-value**	***t*-test** ***p*-value**	**Missing** **(*n*)**
Age	69.4 (6.2)	−0.418	0.0001		0
Fat%^a^	31.4 (9.4)	0.174	0.202		3
BMI^a^	28.0 (4.9)	0.172	0.127		3
Sex				0.747	0
Male	34 (45.9%)				
Female	40 (54.1%)				
Smoking status				0.767	
Non-smoker	71 (95.9%)				
Smoker	3 (4.1%)				
Education				All > 0.259	1
Higher education	57 (78.1%)				
Secondary education	14 (19.2%)				
Basic education	2 (2.7%)				

*^a^adjusted for age and sex. Continuous parameters are expressed as mean values (SD); categorical parameters are expressed as n (%). BMI, body mass index; MoCA, Montreal Cognitive Assessment*.

### The Relation Between Age, Body Fat Percentage and Cognition, and Brain GMV and Peripheral Inflammation

Older age was significantly correlated with lower brain volumes in all regions of interest (all *p* < 0.05). Multiple linear regression, adjusted for age and educational level, showed that higher MoCA scores significantly correlated with higher levels of kynurenine (*p* = 0.027), which is contradictory to what we hypothesized. After adjusting for age and sex, higher fat% was correlated with lower total GMV (*p* = 0.029) and lower left MTC GMV (*p* = 0.031), see Table 3. The correlations with age all survived multiple testing correction with FDR, while the correlations with MoCA and fat% did not remain statistically significant. No significant correlations were found between age or fat% and IL-6 or kynurenine levels, see Supplementary Table 3.

**Table 3:** The effect of age, MoCA, and fat% on brain GMV and peripheral inflammation.

		**β**	***p*-value**
Age	Total GMV	−0.372	0.005
	DPCC GMV	−0.426	0.001
	Left HPC GMV	−0.450	0.0002
	Right HPC GMV	−0.534	0.000005
	Left MTC GMV	−0.320	0.017
	Left SM1 GMV	−0.431	0.0008
	Right DLPFC GMV	−0.377	0.004
MoCA^a^	Kynurenine	0.312	0.027
Fat%^b^	Total GMV	−0.292	0.029
	Left MTC GMV	−0.320	0.031

*Only statistically significant correlations are presented. See Supplementary Table 3 for all results. ^a^adjusted for age and educational level, ^b^adjusted for age and sex. DLPFC, dorsolateral prefrontal cortex; DPCC, dorsal posterior cingulate cortex; GMV, gray matter volume; HPC, hippocampal cortex; mIns, myoinositol; MoCA, Montreal Cognitive Assessment; MTC, medial temporal cortex; o.e., outlier excluded; SM1, primary sensorimotor cortex; tCho, total choline; tCr, total creatine, tNAA, total N-acetyl aspartate*.

### The Relation Between Age, Body Fat Percentage and Cognition, and Neurometabolites

Correlations obtained from regression analyses between age, fat% and cognition and the neurometabolites are depicted in Table 4 and Supplementary Tables 4–6. To assess potential effects of MRS data analysis software, we have compared LCModel and Tarquin for the quantification of neurometabolites.

**Table 4:** Effect of age, MoCA, and fat% on neurometabolites.

		**LCModel**		**Tarquin**	
		***β***	***p*** **-value**	***β***	***p*** **-value**
Age	tCho/tCr DPCC o.e.	0.252	0.044		
	tNAA DPCC			−0.276	0.027
	tCr DPCC	−0.315	0.011	−0.305	0.015
	tCr l HPC			−0.279	0.037
	tNAA l SM1	−0.304	0.013		
	tCr l MTC	0.332	0.013		
MoCA^a^	Glx/tCr l MTC			0.299	0.040
	tCho DPCC			0.322	0.020
Fat% ^b^	tNAA/tCr DPCC			−0.314	0.041
	Glx/tCr l HPC	−0.384	0.019		
	tNAA/tCr l MTC	0.330	0.044	0.348	0.037
	Glx/tCr l MTC			0.388	0.021
	tNAA/tCr l SM1	−0.362	0.016	−0.315	0.041
	tNAA/tCr r DLPFC	−0.338	0.026		
	Glx DPCC			0.353	0.029

*Only statistically significant correlations are presented. See Supplementary Tables 4–6 for all results. ^a^adjusted for age and educational level, ^b^adjusted for age and sex. DLPFC, dorsolateral prefrontal cortex; DPCC, dorsal posterior cingulate cortex; GMV, gray matter volume; HPC, hippocampal cortex; l, left; mIns, myoinositol; MoCA, Montreal Cognitive Assessment; MTC, medial temporal cortex; i.e., after exclusion of an influential outlier; r, right; SM1, primary sensorimotor cortex; tCho, total choline; tCr, total creatine, tNAA, total N-acetyl aspartate*.

A common finding after processing ^1^H-MRS data with LCModel and Tarquin was the significant age-related decrease in tCr DPCC levels. Furthermore, we expect tNAA levels to be lower as a function of age. Even though we did not compare to young adults, we were able to find a decrease of tNAA DPCC (Tarquin) and tNAA left SM1 (LCModel) in association with older age. The age-related decrease in tNAA DPCC levels was also close to significance level for LCModel (*p* = 0.053). No age-related changes in neuroinflammation were discovered. After adjusting for age and educational level, MoCA test results were not related to signs of neurodegeneration or neuroinflammation. Contradictory, higher fat%, adjusted for age and sex, was associated with increased tNAA/tCr levels in left MTC, but decreased tNAA/tCr levels in left SM1. In addition, a negative association was found between fat% and tNAA/tCr DPCC (Tarquin) and tNAA/tCr right DLPFC (LCModel). Also, Glx level changes were inconsistent. Results after processing with LCModel showed higher fat% correlates with lower Glx/tCr in left HPC, while after processing with Tarquin higher fat% correlated with higher levels of Glx/tCr in left MTC and Glx in DPCC. Here, the discordance may be explained by the very weak agreement of Glx concentrations between LCModel and Tarquin results. None of the significant *p*-values survived FDR correction for multiple testing.

### The Relation Between Peripheral Inflammatory Markers, and Brain GMV and Neurometabolites Adjusted for Age, Sex, and Body Fat Percentage

Multiple regression analysis adjusted for age, sex, and fat% and after exclusion of an outlier in IL-6 found no associations between the inflammatory serum markers and brain GMV, see Supplementary Table 7 for all results. Correlations obtained from multiple regression analysis between the inflammatory serum markers and neurometabolites are depicted in Table 5 and Supplementary Tables 8–10.

**Table 5:** The effect of inflammatory blood markers adjusted for age, sex and fat% on neurometabolites.

		**LCModel**		**Tarquin**	
		***β***	***p*** **-value**	***β***	***p*** **-value**
Log IL-6 o.e.	Glx/tCr l HPC			0.317	0.033
	tNAA/tCr l MTC	−0.407	0.011		
	tCho/tCr l MTC	0.442	0.007		
	Glx/tCr l MTC			0.425	0.006
	Glx l HPC			0.306	0.043
	tNAA l MTC	−0.486	0.002		
	Glx l MTC			0.433	0.005
	tNAA l SM1	0.403	0.002		
	Glx rDLPFC			0.339	0.022
Kynurenine	tNAA/tCr l MTC	−0.312	0.026		
	tCho/tCr l MTC	0.418	0.003		
	mIns/tCr l SM1			0.317	0.012
	tNAA/mIns l SM1			−0.255	0.039
	tCho/tCr rDLPFC	0.297	0.020		
	mIns DPCC			0.314	0.016
	tNAA l HPC			−0.317	0.022
	tCr l HPC			−0.379	0.005
	tNAA l MTC	−0.364	0.007		
	tCho l MTC	0.275	0.049		
	tCho l SM1	0.319	0.015		
	mIns l SM1	0.278	0.020	0.375	0.005
	tCho rDLPFC	0.436	0.001	0.386	0.002
	mIns rDLPFC	0.302	0.014		

*Only statistically significant correlations are presented. See Supplementary Tables 8–10 for all results, including results before exclusion of the outlier in Log IL-6. Multiple linear regression analysis was used adjusted for age, sex and fat%. DLPFC, dorsolateral prefrontal cortex; DPCC, dorsal posterior cingulate cortex; Glx, glutamate–glutamine complex; GMV, gray matter volume; HPC, hippocampal cortex; IL-6, interleukin-6; l, left; mIns, myoinositol; MoCA, Montreal Cognitive Assessment; MTC, medial temporal cortex; o.e., outlier excluded; r, right; SM1, primary sensorimotor cortex; tCho, total choline; tCr, total creatine, tNAA, total N-acetyl aspartate*.

The only concordant significant association between peripheral inflammatory markers and neurometabolites for results after processing ^1^H-MRS data with LCModel compared to Tarquin, was the positive association between serum kynurenine levels and mIns levels in the left SM1 and tCho levels in the right DLPFC. Serum kynurenine was associated with signs of neuroinflammation (i.e., the concomitant increase in tCho and mIns) in both left SM1 and right DLPFC when considering LCModel results. After processing ^1^H-MRS data with Tarquin, the positive association between serum kynurenine and tCho left SM1 (*p* = 0.204) and mIns right DLPFC (*p* = 0.063) was non-significant. The increase in tCho in the right DLPFC (LCModel and Tarquin) and the increase in mIns left SM1 (Tarquin) were the only significant associations between neurometabolites and kynurenine that survived the FDR procedure to correct for multiple testing. In addition, although not surviving multiple testing correction, we found a significant positive association between serum kynurenine and mIns concentrations in the DPCC (Tarquin), which was accompanied with a non-significant positive association with tCho DPCC (*p* = 0.162). For LCModel results both the association between serum kynurenine and mIns DPCC (*p* = 0.849) and tCho DPCC (*p* = 0.286) was positive but non-significant. Of interest was also the negative association between serum kynurenine levels and tNAA/mIns concentrations in left SM1 (Tarquin), tNAA left HPC (Tarquin), and tNAA left MTC (LCModel), which may indicate neurodegeneration. When looking at the same results from the other ^1^H-MRS post-processing method where these findings were not significant, we found similar trends for serum kynurenine and tNAA/mIns in left SM1 (with LCModel *p* = 0.128), tNAA left HPC (with LCModel *p* = 0.076), and tNAA left MTC (with Tarquin *p* = 0.053).

Associations between serum IL-6 levels and the neurometabolites were discordant between and within post-processing methods LCModel and Tarquin. While tNAA levels in the left MTC were decreased, tNAA levels in left SM1 were increased in association with high IL-6 levels after processing with LCModel, while results from Tarquin showed the same trends but not reaching significance. These LCModel results were also the only two associations between IL-6 and neurometabolites that survived the FDR multiple testing correction. After processing with Tarquin, we found only an increase in Glx levels related to high IL-6 levels. This was significant for left HPC, left MTC and right DLPFC, and close to significant for DPCC (*p* = 0.062), but none of them survived correction for multiple testing.

## Discussion

We examined the association between serum inflammatory factors and neurometabolic, brain volume, and cognitive changes, taking into account the effect of age, sex, and fat%. Our results showed that the elevated levels of serum kynurenine were associated with signs of neuroinflammation [defined as a concomitant increase in tCho and mIns levels (69)] in the left SM1 and right DLPFC after ^1^H-MRS post-analysis with the LCModel. After an analysis with Tarquin, only a significant increase in mIns left SM1 and tCho right DLPFC was found. After correction for multiple testing, the positive association between serum kynurenine and tCho right DLPFC (LCModel and Tarquin) and mIns left SM1 (Tarquin) remained statistically significant. Furthermore, we found an association between higher kynurenine levels and increases in mIns in the DPCC (Tarquin). This elevation of mIns associated with only a trend of increased tCho levels, as seen in DPCC, most likely also represents neuroinflammation, as increased levels of mIns are believed to represent glial proliferation or an increase in glial cell size (74). Moreover, it has been suggested that elevated levels of mIns or mIns/tCr are an early event in the course toward Alzheimer's dementia and can precede tNAA reduction (75). The previous studies have also reported a positive correlation between pro-inflammatory factors, IL-8 (45) or CRP (76) and hippocampal mIns levels after processing the ^1^H-MRS data with the LCModel (45) or in a study where the ^1^H-MRS post-processing analytical method was not defined (76). In our study, we did not find a significant relationship between one of the measured pro-inflammatory factors and mIns levels in the hippocampus with either of the post-processing software packages. An interesting finding was the association between peripheral inflammation, as marked by high serum levels of kynurenine, and decreases in tNAA/mIns levels in left SM1 (Tarquin). As tNAA/mIns is considered to reflect the combination of decreased neural integrity and increased neuroinflammation, which may indicate neurodegeneration and was previously found to be related to an increased risk of developing clinical Alzheimer's disease (72). Furthermore, an increase in tCho or tCho/tCr was found in the left MTC in association with elevated levels of serum IL-6 and kynurenine in LCModel results. In this case, there was no associated trend of increased mIns. However, the left MTC did present significant decreases in tNAA levels in association with increased levels of serum IL-6 or kynurenine (LCModel). This elevation of tCho, which not coincides with an elevation in mIns, is difficult to interpret, as tCho is a marker of increased synthesis and degradation of cellular membranes, which is not only seen in local inflammation, but also in several other neurological disorders, including cerebral infarctions, multiple sclerosis, and malignant tumors (77).

An interesting finding that was only seen in the results obtained after processing the ^1^H-MRS data with Tarquin was the elevation of Glx and Glx/tCr levels in left HPC and left MTC in association with higher levels of serum IL-6, adjusted for age, sex, and fat%. Notably, also in the DPCC and right DLPFC, Glx and Glx/tCr levels were close to being significantly positively associated with IL-6 levels, see Supplementary Table 9. These findings are in line with observations in a recent study by Ho et al. (78) who used the LCModel post-processing software and reported an association between increases in circulating IL-6 and increased concentrations of glutamate in the anterior cingulate cortex in adolescents with depression. Elevated IL-6 levels have previously also been linked to depression in older adults (79) and recent studies also found increases in Glx in the anterior or posterior cingulate cortex with LCModel in adolescents with suicidal ideation or in cognitively impaired adults (79–81). It has been suggested that inflammatory cytokines can induce an excessive release of glutamate, which results in oligodendrocyte excitotoxicity. Over time, oligodendrocyte excitotoxicity leads to white matter damage and cell apoptosis (78, 82, 83). In contrast, a meta-analysis showed that Glx levels were significantly decreased in the medial frontal cortex in depressed subjects receiving antidepressant medication (84). Also in mild cognitive impairment and Alzheimer's disease, most studies showed decreases of brain glutamate and Glx (75, 85). Furthermore, most studies have reported a decrease in brain glutamate levels with older age, while glutamine was found to increase and Glx to change inconsistently across studies (86, 87).

The discordance between studies and absence of an association between Glx and serum IL-6 levels in the LCModel in our study can partly be explained by the very low agreement between Tarquin and LCModel results concerning Glx. For all other neurometabolites we found an overall moderate-to-high reliability between the two post-processing software packages. This is in line with other studies who also reported that neurometabolites with prominent singlets (like tNAA and tCho) show better agreement between linear-combination modeling algorithms than signals of lower intensity (like mIns and Glx) (65, 88–91). Based on these findings, it is advised to be careful when interpreting results from studies who use only one software package, especially when sample sizes are small and specifically when findings concern Glx. It has been advocated that reporting results of more than one algorithm may improve the power of ^1^H-MRS studies (65).

In this study, serum IL-6 and kynurenine levels were not associated with older age, fat%, or cognitive decline. We even found a positive association between serum kynurenine and cognitive function. These findings are in contrast to findings from the previous studies. Considering IL-6, chronic elevations were reported in association with older age (47) and obesity (92, 93). In addition, high levels of peripheral IL-6 negatively affected memory and learning (94) and correlated with poor overall cognitive performance (95, 96). Moreover, high basal IL-6 levels were also associated with an increased risk of future age-related cognitive decline (48, 97). In our study, IL-6 levels inversely correlated with hippocampal GMV (94, 98), which could only be found in the right HPC before exclusion of an outlier in IL-6, see Supplementary Table 7. Based on the findings from existing literature, it has been suggested that high levels of IL-6 may increase the risk of developing neurodegenerative diseases (99). However, our results suggest that serum kynurenine, despite its contradictory positive association with cognitive functioning, would be a better predictor of neuroinflammation and neurodegeneration than IL-6. Of note, IL-6, though widely used as a marker of inflammation, has been suggested to be not only involved in pro-inflammatory processes, but to act as a regulator of both pro- and anti-inflammatory activity (49). For example, a study in healthy humans showed that the administration of IL-6 before an infusion with endotoxin abolished the increase in plasma levels of the pro-inflammatory factor TNF-α (100). In contrast, kynurenine is considered a more robust marker of inflammation, as several pro-inflammatory cytokines, like CRP, IL-1β, IL-6, IL-8, TNF-α, and IFN-γ, were shown to enhance the activation of indoleamine 2,3-dioxygenase, increasing kynurenine levels, while the anti-inflammatory cytokines IL-4 and IL-10 counteracted this increase in kynurenine levels (50–54). Furthermore, kynurenine has previously been linked to obesity (57), cognitive aging (58), psychiatric disorders (54, 55, 59), brain volume loss (59), neurodegenerative disorders (60), and central nervous system injury (50). Taking into account the vast amount of evidence in the recent literature and considering our own results, we propose that kynurenine can be used as a generic peripheral inflammatory marker that is associated with neuroinflammation and neurodegeneration. However, still more research needs to confirm the role of elevated kynurenine levels in age-related cognitive decline and neurodegenerative diseases.

In conclusion, serum kynurenine levels were positively associated with signs of neuroinflammation, specifically in the DPCC, left SM1, and right DLPFC, and the signs of neurodegeneration, specifically in the left HPC, left MT,C and left SM1, after adjusting for age, sex, and fat%. Interestingly, higher levels of IL-6 were associated with an elevation of Glx levels in left HPC, left MTC, and right DLPFC, but only following ^1^H-MRS post-processing analysis with Tarquin. Of note, the agreement between Tarquin and LCModel results was moderate-to-strong for tNAA, tCho, mIns and tCr, but weak to very weak for Glx in all regions of interest. At last, our results did not find a link between the peripheral inflammatory markers kynurenine or IL-6 and older age, fat%, or cognitive decline. It should be noted that our results are exploratory and should be interpreted with caution due to multiple testing. After correcting for multiple testing with FDR analysis, the positive association between serum kynurenine and tCho right DLPFC (LCModel and Tarquin) and mIns left SM1 (Tarquin) remained statistically significant. Furthermore, a discordant finding of an association between higher IL-6 levels and lower tNAA levels in the left MTC, but higher tNAA levels in left SM1 survived the correction for multiple testing. At last, the negative associations between age and MoCA score and between age and GMV in all regions of interest remained statistically significant after the FDR procedure. Further analyses are needed to specifically assess the relationship between increases of peripheral inflammatory factors and markers of neuroinflammation in older adults and obesity and investigate their role in cognitive decline.

## Data Availability Statement

The raw data supporting the conclusions of this article will be made available by the authors, without undue reservation.

## Ethics Statement

The studies involving human participants were reviewed and approved by Medical Ethics Committee for Biomedical Research of the Lithuanian Sports University (No. BE-10-7). The patients/participants provided their written informed consent to participate in this study.

## Author Contributions

WV, SK, VC, OL, and NM contributed to conception and design of the study. WV, SK, SS, MŠ, KV, RG, MK, VC, OL, and NM were involved in data collection and/or analysis. WV and KP performed the statistical analysis. WV wrote the first draft of the manuscript. SK, MŠ, MK, and UH wrote sections of the manuscript. OL prepared the figures. RG, OL, JV, and NM had a role in supervision. All authors contributed to manuscript revision, read, and approved the submitted version.

## Funding

This work was supported by the Research Council of Lithuania (Grant Number S-SEN-20-5). The sponsor was not involved in study design; collection, analysis, and interpretation of the data; in writing of the report; nor in the decision to submit the article for publication.

## Conflict of Interest

The authors declare that the research was conducted in the absence of any commercial or financial relationships that could be construed as a potential conflict of interest.

## Publisher's Note

All claims expressed in this article are solely those of the authors and do not necessarily represent those of their affiliated organizations, or those of the publisher, the editors and the reviewers. Any product that may be evaluated in this article, or claim that may be made by its manufacturer, is not guaranteed or endorsed by the publisher.

## Abbreviations

^1^H-MRS: proton magnetic resonance spectroscopy
BMI: body mass index
CRP: C-Reactive protein
DLPFC: dorsolateral prefrontal cortex
DPCC: dorsal posterior cingulate cortex
FDR: false discovery rate
Fat%: body fat percentage
Glx: glutamate–glutamine complex
GMV: gray matter volume
HPC: hippocampal cortex
ICC: intraclass correlation coefficient
IL-6: interleukin-6
IL-8: interleukin-8
mIns: myoinositol
MoCA: Montreal Cognitive Assessment
MTC: medial temporal cortex
SM1: primary sensorimotor cortex
tCho: total choline
tCr: total creatine
tNAA: total N-acetyl aspartate
TNF-α: tumor necrosis factor-α.
