# Supplementary material for: Inflammatory Blood Biomarker Kynurenine Is Linked With Elevated Neuroinflammation and Neurodegeneration in Older Adults: Evidence From Two 1H-MRS Post-Processing Analysis Methods
Source: Front Psychiatry. 2022 Apr 11;13:859772. doi: 10.3389/fpsyt.2022.859772 (PMC9035828; doi:10.3389/fpsyt.2022.859772)
Supplement: Supplementary file 1 [file Data_Sheet_1.docx]

**Supplementary tables**

| **Supplementary table 1.** Agreement between LCModel and non-lipid filtered Tarquin ^1^H-MRS post-processing software packages for right dorsolateral prefrontal cortex neurometabolite levels | | | | | | |
| --- | --- | --- | --- | --- | --- | --- |
|  |  | tNAA | tCho | Glx | mIns | tCr |
| Right DLPFC | R-value  ICC | 0.449***  0.395*** | 0.774***  0.641*** | 0.214  0.061* | 0.747***  0.491*** | 0.523***  0.193*** |
| Pearson R and ICC values are presented for the correlation between Tarquin and LCModel measurements. * p < 0.05, ** p < 0.01, *** p < 0.001; Abbreviations: DLPFC, dorsolateral prefrontal cortex; DPCC, dorsal posterior cingulate cortex; Glx, glutamate-glutamine complex; HPC, hippocampal cortex; ICC, intraclass correlation coefficient; mIns, myoinositol; MTC, medial temporal cortex; SM1, primary sensorimotor cortex; tCho, total choline; tCr, total creatine, tNAA, total N-acetyl aspartate | | | | | | |

| **Supplementary table 2.** Number of good quality measurements for MRI and ^1^H-MRS, total n = 74 | | | |
| --- | --- | --- | --- |
| MRI | GMV | 55 |  |
|  |  |  |  |
|  |  | LCModel | Tarquin |
| ^1^H-MRS | DPCC | 65 | 65 |
|  | Left HPC | 61 | 54 |
|  | Left MTC | 56 | 59 |
|  | Left SM1 | 66 | 66 |
|  | Right DLPFC | 67 | 64 |
| The values presented are the number (n) of subjects with complete, good quality data for all MRI GMV measurements or ^1^H-MRS measurements for each of the voxels and after processing with LCModel or with Tarquin. Abbreviations: ^1^H-MRS, proton magnetic resonance spectroscopy; DLPFC, dorsolateral prefrontal cortex; DPCC, dorsal posterior cingulate cortex; GMV, gray matter volume; HPC, hippocampal cortex; MRI, magnetic resonance imaging; MTC, medial temporal cortex; SM1, primary sensorimotor cortex. | | | |

| **Supplementary table 3.** The effect of age, MoCA and fat % on brain gray matter volume and peripheral inflammation | | | | |
| --- | --- | --- | --- | --- |
|  |  | B (95% CI) | β | p-value |
| Age | MoCA | -0.193 (-0.292,-0.092) | -0.418 | 0.0002*** |
|  | Log IL-6 | 0.005 (-0.020,0.031) | 0.053 | 0.688 |
|  | Log IL-6 o.e. | 0.002 (-0.019, 0.023) | 0.021 | 0.875 |
|  | Kynurenine | 9.381 (-18.869, 37.630) | 0.083 | 0.509 |
|  | Total GMV | -3355.357 (-5665.423, -1045.291) | -0.372 | 0.005** |
|  | DPCC GMV | -60.538 (-95.968, -25.108) | -0.426 | 0.001** |
|  | Left HPC GMV | -28.269 (-42.507, -14.031) | -0.450 | 0.0002*** |
|  | Right HPC GMV | -30.922 (-43.348, -18.496) | -0.534 | 0.000005*** |
|  | Left MTC GMV | -77.093 (-139.954, -14.232) | -0.320 | 0.017* |
|  | Left SM1 GMV | -196.786 (-308.178, -85.398) | -0.431 | 0.0008*** |
|  | Right DLPFC GMV | -174.658 (-290.579, -58.737) | -0.377 | 0.004** |
| MoCA^a^ | Log IL-6 | 0.038 (-0.022, 0.099) | 0.190 | 0.211 |
|  | Log IL-6 o.e. | 0.022 (-0.028, 0.072) | 0.135 | 0.379 |
|  | Kynurenine | 76.838 (9.080, 144.595) | 0.312 | 0.027* |
|  | Total GMV | 3455.112 (-1873.251, 8783.475) | 0.185 | 0.199 |
|  | DPCC GMV | 31.690 (-50.270, 113.650) | 0.108 | 0.441 |
|  | Left HPC GMV | 26.143 (-9.067, 61.353) | 0.188 | 0.143 |
|  | Right HPC GMV | 20.792 (-10.230, 51.814) | 0.161 | 0.185 |
|  | Left MTC GMV | 117.924 (-25.252, 261.101) | 0.236 | 0.104 |
|  | Left SM1 GMV | 162.507 (-88.706, 413.721) | 0.178 | 0.200 |
|  | Right DLPFC GMV | 229.293 (-29.933, 488.520) | 0.245 | 0.082 |
| Fat %^b^ | MoCA | 0.052 (-0.028, 0.132) | 0.174 | 0.202 |
|  | Log IL-6 | -0.008 (-0.031, 0.014) | -0.137 | 0.447 |
|  | Log IL-6 o.e. | -0.008 (-0.028, 0.011) | -0.158 | 0.388 |
|  | Kynurenine | 12.064 (-12.656, 36.783) | 0.151 | 0.333 |
|  | Total GMV | -1629.150 (-3083.135, -175.165) | -0.292 | 0.029* |
|  | DPCC GMV | -5.120 (-29.392, 19.152) | -0.058 | 0.674 |
|  | Left HPC GMV | -6.534 (-17.518, 4.451) | -0.162 | 0.239 |
|  | Right HPC GMV | -8.177 (-17.540, 1.186) | -0.215 | 0.086 |
|  | Left MTC GMV | -47.490 (-90.486, -4.494) | -0.320 | 0.031* |
|  | Left SM1 GMV | -49.874 (-132.977, 33.229) | -0.169 | 0.234 |
|  | Right DLPFC GMV | -19.265 (-103.596, 65.066) | -0.066 | 0.649 |
| * p < 0.05, ** p < 0.01, *** p < 0.001; ^a^ adjusted for age and educational level, ^b^ adjusted for age and gender. Abbreviations: DLPFC, dorsolateral prefrontal cortex; DPCC, dorsal posterior cingulate cortex; GMV, gray matter volume; HPC, hippocampal cortex; IL-6, interleukin-6; mIns, myoinositol; MoCA, Montreal Cognitive Assessment; MTC, medial temporal cortex; o.e., influential outlier excluded; SM1, primary sensorimotor cortex; tCho, total choline; tCr, total creatine, tNAA, total N-acetyl aspartate | | | | |

| **Supplementary table 4.** Effect of age on brain neurometabolites | | | | | |
| --- | --- | --- | --- | --- | --- |
|  | | LCModel | | Tarquin | |
|  |  | β | p-value | β | p-value |
| Age | tNAA/tCr DPCC | 0.076 | 0.549 | -0.002 | 0.986 |
|  | tCho/tCr DPCC | 0.218 | 0.080 | 0.075 | 0.552 |
|  | tCho/tCr DPCC o.e. | 0.252 | 0.044* | 0.067 | 0.597 |
|  | Glx/tCr DPCC | 0.045 | 0.720 | 0.052 | 0.683 |
|  | Glx/tCr DPCC o.e. | 0.031 | 0.810 | 0.061 | 0.630 |
|  | mIns/tCr DPCC | 0.020 | 0.872 | 0.064 | 0.614 |
|  | tNAA/mIns DPCC | 0.016 | 0.898 | -0.036 | 0.773 |
|  | tNAA/tCr l HPC | -0.057 | 0.664 | -0.051 | 0.708 |
|  | tCho/tCr l HPC | -0.070 | 0.592 | -0.066 | 0.630 |
|  | Glx/tCr l HPC | 0.169 | 0.193 | 0.151 | 0.271 |
|  | Glx/tCr l HPC o.e. |  |  | 0.156 | 0.276 |
|  | mIns/tCr l HPC | 0.139 | 0.285 | -0.053 | 0.698 |
|  | tNAA/mIns l HPC | -0.155 | 0.233 | 0.000 | 0.999 |
|  | tNAA/tCr l SM1 | -0.184 | 0.139 | -0.090 | 0.478 |
|  | tCho/tCr l SM1 | -0.126 | 0.314 | -0.177 | 0.159 |
|  | Glx/tCr l SM1 | -0.110 | 0.378 | -0.119 | 0.305 |
|  | mIns/tCr l SM1 | -0.072 | 0.565 | -0.088 | -0.700 |
|  | tNAA/mIns l SM1 | -0.018 | 0.888 | 0.089 | 0.481 |
|  | tNAA/tCr l MTC | -0.093 | 0.499 | -0.117 | 0.378 |
|  | tCho/tCr l MTC | -0.085 | 0.537 | -0.040 | 0.766 |
|  | Glx/tCr l MTC | -0.203 | 0.137 | 0.085 | 0.525 |
|  | Glx/tCr l MTC o.e. |  |  | 0.009 | 0.945 |
|  | mIns/tCr l MTC | -0.129 | 0.349 | 0.172 | 0.194 |
|  | mIns/tCr l MTC o.e. |  |  | 0.094 | 0.482 |
|  | tNAA/mIns l MTC | 0.089 | 0.520 | 0.006 | 0.963 |
|  | tNAA/tCr r DLPFC | -0.048 | 0.697 | 0.008 | 0.950 |
|  | tCho/tCr r DLPFC | -0.096 | 0.440 | -0.127 | 0.316 |
|  | Glx/tCr r DLPFC | -0.048 | 0.697 | -0.031 | 0.819 |
|  | mIns/tCr r DLPFC | -0.170 | 0.168 | -0.121 | 0.339 |
|  | tNAA/mIns r DLPFC | 0.137 | 0.270 | 0.117 | 0.357 |
|  | tNAA DPCC | -0.242 | 0.053 | -0.276 | 0.027* |
|  | tCho DPCC | -0.036 | 0.775 | -0.129 | 0.313 |
|  | tCho DPCC o.e. | -0.052 | 0.682 |  |  |
|  | Glx DPCC | -0.129 | 0.304 | -0.030 | 0.818 |
|  | mIns DPCC | -0.151 | 0.231 | -0.122 | 0.339 |
|  | tCr DPCC | -0.315 | 0.011* | -0.305 | 0.015* |
|  | tNAA l HPC | -0.224 | 0.082 | -0.196 | 0.144 |
|  | tCho l HPC | -0.231 | 0.073 | -0.238 | 0.077 |
|  | Glx l HPC | 0.015 | 0.912 | 0.122 | 0.376 |
|  | Glx l HPC o.e. |  |  | 0.070 | 0.615 |
|  | mIns l HPC | -0.026 | 0.840 | -0.194 | 0.153 |
|  | mIns l HPC o.e. |  |  | -0.103 | 0.453 |
|  | tCr l HPC | -0.164 | 0.206 | -0.279 | 0.037* |
|  | tCr l HPC o.e. | -0.134 | 0.306 |  |  |
|  | tNAA l SM1 | -0.304 | 0.013* | -0.133 | 0.287 |
|  | tCho l SM1 | -0.194 | 0.119 | -0.207 | 0.096 |
|  | Glx l SM1 | -0.158 | 0.206 | -0.133 | 0.291 |
|  | mIns l SM1 | -0.132 | 0.292 | -0.100 | 0.426 |
|  | tCr l SM1 | -0.156 | 0.211 | -0.060 | 0.633 |
|  | tNAA l MTC | 0.190 | 0.165 | -0.092 | 0.489 |
|  | tCho l MTC | 0.214 | 0.117 | -0.016 | 0.906 |
|  | tCho l MTC o.e. |  |  | 0.058 | 0.664 |
|  | Glx l MTC | 0.004 | 0.977 | 0.063 | 0.637 |
|  | mIns l MTC | 0.056 | 0.685 | 0.154 | 0.245 |
|  | tCr l MTC | 0.332 | 0.013* | 0.016 | 0.904 |
|  | tCr l MTC o.e. |  |  | 0.087 | 0.515 |
|  | tNAA r DLPFC | -0.114 | 0.364 | -0.041 | 0.750 |
|  | tCho r DLPFC | -0.151 | 0.225 | -0.159 | 0.210 |
|  | Glx r DLPFC | -0.063 | 0.617 | -0.066 | 0.624 |
|  | mIns r DLPFC | -0.191 | 0.125 | -0.146 | 0.249 |
|  | tCr r DLPFC | -0.060 | 0.633 | -0.059 | 0.645 |
| * p < 0.05; Single linear regression analysis for age. Abbreviations: DLPFC, dorsolateral prefrontal cortex; DPCC, dorsal posterior cingulate cortex; GMV, gray matter volume; HPC, hippocampal cortex; l, left; mIns, myoinositol; MTC, medial temporal cortex; o.e., influential outlier excluded; r, right; SM1, primary sensorimotor cortex; tCho, total choline; tCr, total creatine, tNAA, total N-acetyl aspartate. | | | | | |

| **Supplementary table 5.** Effect of MoCA on brain neurometabolites | | | | | |
| --- | --- | --- | --- | --- | --- |
|  |  | LCModel | | Tarquin | |
|  |  | β | p-value | β | p-value |
| MoCA | tNAA/tCr DPCC | -0.174 | 0.227 | -0.220 | 0.121 |
|  | tCho/tCr DPCC | 0.086 | 0.531 | 0.174 | 0.216 |
|  | Glx/tCr DPCC | -0.182 | 0.206 | 0.003 | 0.985 |
|  | mIns/tCr DPCC | 0.068 | 0.634 | 0.106 | 0.456 |
|  | tNAA/mIns DPCC | -0.107 | 0.454 | -0.214 | 0.127 |
|  | tNAA/tCr l HPC | -0.084 | 0.574 | -0.126 | 0.434 |
|  | tCho/tCr l HPC | 0.019 | 0.898 | -0.014 | 0.929 |
|  | Glx/tCr l HPC | 0.044 | 0.769 | -0.001 | 0.997 |
|  | mIns/tCr l HPC | 0.054 | 0.718 | -0.041 | 0.797 |
|  | tNAA/mIns l HPC | -0.123 | 0.404 | -0.040 | 0.801 |
|  | tNAA/tCr l SM1 | -0.130 | 0.350 | -0.028 | 0.841 |
|  | tCho/tCr l SM1 | 0.023 | 0.868 | 0.045 | 0.750 |
|  | Glx/tCr l SM1 | -0.122 | 0.390 | 0.020 | 0.892 |
|  | mIns/tCr l SM1 | -0.043 | 0.760 | -0.049 | 0.731 |
|  | tNAA/mIns l SM1 | -0.038 | 0.788 | 0.016 | 0.909 |
|  | tNAA/tCr l MTC | -0.139 | 0.388 | 0.068 | 0.654 |
|  | tCho/tCr l MTC | 0.257 | 0.107 | 0.282 | 0.062 |
|  | Glx/tCr l MTC | -0.017 | 0.910 | 0.299 | 0.040* |
|  | mIns/tCr l MTC | 0.086 | 0.593 | 0.254 | 0.073 |
|  | tNAA/mIns l MTC | -0.104 | 0.517 | -0.237 | 0.109 |
|  | tNAA/tCr r DLPFC | -0.145 | 0.302 | 0.054 | 0.710 |
|  | tCho/tCr r DLPFC | 0.048 | 0.731 | 0.047 | 0.741 |
|  | Glx/tCr r DLPFC | -0.083 | 0.554 | 0.021 | 0.893 |
|  | mIns/tCr r DLPFC | 0.039 | 0.776 | 0.151 | 0.292 |
|  | tNAA/mIns r DLPFC | -0.108 | 0.436 | -0.091 | 0.527 |
|  | tNAA DPCC | 0.026 | 0.854 | -0.031 | 0.824 |
|  | tCho DPCC | 0.212 | 0.128 | 0.322 | 0.020* |
|  | Glx DPCC | -0.052 | 0.718 | 0.058 | 0.685 |
|  | mIns DPCC | 0.162 | 0.249 | 0.267 | 0.059 |
|  | tCr DPCC | 0.167 | 0.221 | 0.210 | 0.126 |
|  | tNAA l HPC | 0.015 | 0.921 | -0.055 | 0.726 |
|  | tCho l HPC | 0.117 | 0.430 | 0.042 | 0.787 |
|  | Glx l HPC | 0.066 | 0.665 | -0.013 | 0.936 |
|  | mIns l HPC | 0.120 | 0.427 | 0.025 | 0.865 |
|  | tCr l HPC | 0.099 | 0.505 | 0.070 | 0.645 |
|  | tNAA l SM1 | -0.012 | 0.932 | 0.049 | 0.723 |
|  | tCho l SM1 | 0.099 | 0.478 | 0.130 | 0.350 |
|  | Glx l SM1 | -0.060 | 0.668 | 0.047 | 0.739 |
|  | mIns l SM1 | 0.031 | 0.824 | 0.006 | 0.969 |
|  | tCr l SM1 | 0.172 | 0.217 | 0.112 | 0.431 |
|  | tNAA l MTC | -0.273 | 0.082 | -0.077 | 0.616 |
|  | tCho l MTC | 0.091 | 0.564 | 0.099 | 0.524 |
|  | Glx l MTC | -0.151 | 0.336 | 0.211 | 0.154 |
|  | mIns l MTC | 0.008 | 0.962 | 0.216 | 0.122 |
|  | tCr l MTC | -0.197 | 0.189 | -0.199 | 0.196 |
|  | tNAA r DLPFC | 0.106 | 0.454 | 0.129 | 0.366 |
|  | tCho r DLPFC | 0.100 | 0.478 | 0.109 | 0.446 |
|  | Glx r DLPFC | -0.079 | 0.580 | 0.009 | 0.957 |
|  | mIns r DLPFC | 0.061 | 0.658 | 0.203 | 0.153 |
|  | tCr r DLPFC | 0.066 | 0.645 | 0.087 | 0.546 |
| * p < 0.05; Multiple linear regression analysis was used adjusted for age and educational level. Abbreviations: DLPFC, dorsolateral prefrontal cortex; DPCC, dorsal posterior cingulate cortex; GMV, gray matter volume; HPC, hippocampal cortex; l, left; mIns, myoinositol; MoCA, Montreal Cognitive Assessment; MTC, medial temporal cortex; o.e., influential outlier excluded; r, right; SM1, primary sensorimotor cortex; tCho, total choline; tCr, total creatine, tNAA, total N-acetyl aspartate. | | | | | |

| **Supplementary table 6.** Effect of Fat % on brain neurometabolites | | | | | |
| --- | --- | --- | --- | --- | --- |
|  |  | LCModel | | Tarquin | |
|  |  | β | p-value | β | p-value |
| Fat % | tNAA/tCr DPCC | -0.202 | 0.216 | -0.314 | 0.041* |
|  | tCho/tCr DPCC | -0.147 | 0.341 | -0.216 | 0.162 |
|  | Glx/tCr DPCC | 0.159 | 0.331 | 0.255 | 0.101 |
|  | mIns/tCr DPCC | -0.099 | 0.548 | -0.021 | 0.893 |
|  | tNAA/mIns DPCC | 0.085 | 0.606 | -0.178 | 0.247 |
|  | tNAA/tCr l HPC | -0.023 | 0.893 | -0.304 | 0.077 |
|  | tCho/tCr l HPC | -0.090 | 0.583 | -0.081 | 0.637 |
|  | Glx/tCr l HPC | -0.384 | 0.019* | -0.232 | 0.185 |
|  | mIns/tCr l HPC | -0.059 | 0.725 | -0.069 | 0.694 |
|  | tNAA/mIns l HPC | 0.009 | 0.957 | 0.000 | 0.999 |
|  | tNAA/tCr l SM1 | -0.362 | 0.016* | -0.315 | 0.041* |
|  | tCho/tCr l SM1 | -0.093 | 0.551 | -0.131 | 0.395 |
|  | Glx/tCr l SM1 | 0.116 | 0.457 | 0.117 | 0.450 |
|  | mIns/tCr l SM1 | -0.016 | 0.915 | -0.005 | 0.976 |
|  | tNAA/mIns l SM1 | -0.097 | 0.521 | -0.126 | 0.405 |
|  | tNAA/tCr l MTC | 0.330 | 0.044* | 0.348 | 0.037* |
|  | tCho/tCr l MTC | -0.245 | 0.140 | -0.139 | 0.414 |
|  | Glx/tCr l MTC | 0.049 | 0.764 | 0.388 | 0.021* |
|  | mIns/tCr l MTC | 0.091 | 0.575 | 0.218 | 0.179 |
|  | tNAA/mIns l MTC | 0.167 | 0.305 | 0.029 | 0.864 |
|  | tNAA/tCr r DLPFC | -0.338 | 0.026* | -0.241 | 0.118 |
|  | tCho/tCr r DLPFC | -0.029 | 0.850 | -0.109 | 0.477 |
|  | Glx/tCr r DLPFC | 0.084 | 0.587 | -0.161 | 0.328 |
|  | mIns/tCr r DLPFC | 0.140 | 0.354 | 0.069 | 0.661 |
|  | tNAA/mIns r DLPFC | -0.217 | 0.150 | -0.139 | 0.370 |
|  | tNAA DPCC | -0.226 | 0.149 | -0.251 | 0.099 |
|  | tCho DPCC | -0.213 | 0.196 | -0.176 | 0.281 |
|  | Glx DPCC | 0.124 | 0.452 | 0.353 | 0.029* |
|  | mIns DPCC | -0.117 | 0.474 | 0.200 | 0.214 |
|  | tCr DPCC | -0.087 | 0.563 | 0.096 | 0.539 |
|  | tNAA l HPC | 0.181 | 0.268 | -0.139 | 0.404 |
|  | tCho l HPC | 0.143 | 0.387 | 0.102 | 0.561 |
|  | Glx l HPC | -0.227 | 0.175 | -0.217 | 0.238 |
|  | mIns l HPC | 0.120 | 0.480 | 0.102 | 0.568 |
|  | tCr l HPC | 0.243 | 0.138 | 0.135 | 0.428 |
|  | tNAA l SM1 | -0.276 | 0.057 | -0.272 | 0.076 |
|  | tCho l SM1 | -0.052 | 0.734 | -0.113 | 0.457 |
|  | Glx l SM1 | 0.133 | 0.392 | 0.123 | 0.427 |
|  | mIns l SM1 | 0.026 | 0.858 | 0.011 | 0.941 |
|  | tCr l SM1 | 0.103 | 0.504 | 0.056 | 0.721 |
|  | tNAA l MTC | 0.236 | 0.138 | 0.219 | 0.199 |
|  | tCho l MTC | -0.254 | 0.115 | -0.239 | 0.157 |
|  | Glx l MTC | 0.044 | 0.795 | 0.283 | 0.101 |
|  | mIns l MTC | 0.101 | 0.545 | 0.080 | 0.634 |
|  | tCr l MTC | -0.039 | 0.800 | -0.214 | 0.209 |
|  | tNAA r DLPFC | -0.190 | 0.218 | -0.258 | 0.091 |
|  | tCho r DLPFC | 0.048 | 0.754 | -0.119 | 0.432 |
|  | Glx r DLPFC | 0.119 | 0.445 | -0.144 | 0.382 |
|  | mIns r DLPFC | 0.212 | 0.158 | 0.064 | 0.680 |
|  | tCr r DLPFC | 0.140 | 0.371 | -0.001 | 0.995 |
| * p < 0.05; Multiple linear regression analysis was used adjusted for age and gender. Abbreviations: DLPFC, dorsolateral prefrontal cortex; DPCC, dorsal posterior cingulate cortex; GMV, gray matter volume; HPC, hippocampal cortex; l, left; mIns, myoinositol; MTC, medial temporal cortex; o.e., influential outlier excluded; r, right; SM1, primary sensorimotor cortex; tCho, total choline; tCr, total creatine, tNAA, total N-acetyl aspartate. | | | | | |

| **Supplementary table 7.** The effect of IL-6 and kynurenine on brain gray matter volume | | | |
| --- | --- | --- | --- |
|  |  | β | p-value |
| Log IL-6 | Total GMV | 0.127 | 0.276 |
|  | DPCC GMV | 0.088 | 0.465 |
|  | Left HPC GMV | -0.174 | 0.159 |
|  | Right HPC GMV | -0.256 | 0.017* |
|  | Left MTC GMV | 0.056 | 0.680 |
|  | Left SM1 GMV | -0.015 | 0.901 |
|  | Right DLPFC GMV | 0.203 | 0.114 |
| Log IL-6 o.e. | Total GMV | 0.092 | 0.431 |
|  | DPCC GMV | 0.112 | 0.361 |
|  | Left HPC GMV | -0.109 | 0.384 |
|  | Right HPC GMV | -0.195 | 0.076 |
|  | Left MTC GMV | -0.023 | 0.865 |
|  | Left SM1 GMV | -0.043 | 0.725 |
|  | Right DLPFC GMV | 0.197 | 0.126 |
| Kynurenine | Total GMV | 0.065 | 0.549 |
|  | DPCC GMV | -0.067 | 0.561 |
|  | Left HPC GMV | -0.079 | 0.497 |
|  | Right HPC GMV | 0.042 | 0.688 |
|  | Left MTC GMV | -0.040 | 0.752 |
|  | Left SM1 GMV | 0.003 | 0.981 |
|  | Right DLPFC GMV | 0.171 | 0.163 |
| * p < 0.05, ** p < 0.01, *** p < 0.001; Multiple linear regression analysis was used adjusted for age, gender and fat%. Abbreviations: DLPFC, dorsolateral prefrontal cortex; DPCC, dorsal posterior cingulate cortex; GMV, gray matter volume; HPC, hippocampal cortex; IL-6, interleukin-6; mIns, myoinositol; MTC, medial temporal cortex; o.e., influential outlier excluded; SM1, primary sensorimotor cortex; tCho, total choline; tCr, total creatine, tNAA, total N-acetyl aspartate | | | |

| **Supplementary table 8.** The effect of inflammatory blood markers adjusted for age, gender and fat% on brain neurometabolites | | | | | |
| --- | --- | --- | --- | --- | --- |
|  | | LCModel | | Tarquin | |
|  |  | β | p-value | β | p-value |
| Log IL-6 | tNAA/tCr DPCC | -0.150 | 0.293 | -0.096 | 0.496 |
|  | tCho/tCr DPCC | 0.020 | 0.880 | -0.116 | 0.424 |
|  | Glx/tCr DPCC | 0.000 | 0.999 | 0.358 | 0.013* |
|  | mIns/tCr DPCC | -0.156 | 0.286 | 0.179 | 0.215 |
|  | tNAA/mIns DPCC | 0.206 | 0.160 | -0.205 | 0.152 |
|  | tNAA/tCr l HPC | -0.064 | 0.680 | -0.230 | 0.136 |
|  | tCho/tCr l HPC | -0.173 | 0.238 | -0.122 | 0.441 |
|  | Glx/tCr l HPC | -0.060 | 0.677 | 0.567 | 0.00008* |
|  | mIns/tCr l HPC | -0.168 | 0.270 | 0.331 | 0.036* |
|  | tNAA/mIns l HPC | 0.127 | 0.406 | -0.409 | 0.008* |
|  | tNAA/tCr l SM1 | 0.160 | 0.250 | 0.098 | 0.487 |
|  | tCho/tCr l SM1 | -0.023 | 0.875 | -0.081 | 0.579 |
|  | Glx/tCr l SM1 | 0.064 | 0.659 | 0.098 | 0.510 |
|  | mIns/tCr l SM1 | 0.038 | 0.776 | 0.037 | 0.786 |
|  | tNAA/mIns l SM1 | 0.052 | 0.711 | -0.001 | 0.991 |
|  | tNAA/tCr l MTC | -0.362 | 0.023* | -0.074 | 0.635 |
|  | tCho/tCr l MTC | 0.294 | 0.076 | 0.086 | 0.597 |
|  | Glx/tCr l MTC | -0.053 | 0.745 | 0.341 | 0.031* |
|  | mIns/tCr l MTC | -0.099 | 0.522 | 0.052 | 0.744 |
|  | tNAA/mIns l MTC | -0.024 | 0.877 | -0.006 | 0.969 |
|  | tNAA/tCr r DLPFC | 0.185 | 0.171 | 0.167 | 0.255 |
|  | tCho/tCr r DLPFC | 0.037 | 0.796 | 0.001 | 0.996 |
|  | Glx/tCr r DLPFC | 0.257 | 0.075 | 0.292 | 0.052 |
|  | mIns/tCr r DLPFC | 0.247 | 0.067 | 0.092 | 0.528 |
|  | tNAA/mIns r DLPFC | -0.135 | 0.316 | -0.018 | 0.900 |
|  | tNAA DPCC | -0.107 | 0.442 | -0.065 | 0.646 |
|  | tCho DPCC | 0.005 | 0.975 | -0.107 | 0.479 |
|  | Glx DPCC | 0.012 | 0.932 | 0.344 | 0.015* |
|  | mIns DPCC | -0.135 | 0.354 | 0.140 | 0.330 |
|  | tCr DPCC | -0.001 | 0.994 | 0.024 | 0.866 |
|  | tNAA l HPC | -0.113 | 0.428 | -0.279 | 0.059 |
|  | tCho l HPC | -0.224 | 0.132 | -0.161 | 0.299 |
|  | Glx l HPC | -0.087 | 0.556 | 0.505 | 0.001* |
|  | mIns l HPC | -0.177 | 0.245 | 0.260 | 0.090 |
|  | tCr l HPC | -0.053 | 0.719 | -0.117 | 0.422 |
|  | tNAA l SM1 | 0.362 | 0.005* | 0.187 | 0.188 |
|  | tCho l SM1 | 0.104 | 0.472 | 0.052 | 0.720 |
|  | Glx l SM1 | 0.145 | 0.307 | 0.103 | 0.481 |
|  | mIns l SM1 | 0.149 | 0.245 | 0.155 | 0.240 |
|  | tCr l SM1 | 0.277 | 0.046* | 0.116 | 0.422 |
|  | tNAA l MTC | -0.549 | 0.0003* | -0.253 | 0.106 |
|  | tCho l MTC | 0.038 | 0.815 | -0.024 | 0.880 |
|  | Glx l MTC | -0.242 | 0.150 | 0.295 | 0.063 |
|  | mIns l MTC | -0.270 | 0.100 | -0.044 | 0.782 |
|  | tCr l MTC | -0.275 | 0.075 | -0.165 | 0.307 |
|  | tNAA r DLPFC | 0.220 | 0.123 | 0.288 | 0.043* |
|  | tCho r DLPFC | 0.063 | 0.662 | 0.090 | 0.538 |
|  | Glx r DLPFC | 0.224 | 0.127 | 0.339 | 0.022* |
|  | mIns r DLPFC | 0.181 | 0.180 | 0.169 | 0.248 |
|  | tCr r DLPFC | 0.051 | 0.729 | 0.162 | 0.290 |
| * p < 0.05; Multiple linear regression analysis was used adjusted for age, gender and fat%. Abbreviations: DLPFC, dorsolateral prefrontal cortex; DPCC, dorsal posterior cingulate cortex; Glx, glutamate-glutamine complex; GMV, gray matter volume; HPC, hippocampal cortex; IL-6, interleukin-6; l, left; mIns, myoinositol; MTC, medial temporal cortex; o.e., outlier excluded; r, right; SM1, primary sensorimotor cortex; tCho, total choline; tCr, total creatine, tNAA, total N-acetyl aspartate | | | | | |

| **Supplementary table 9.** The effect of inflammatory blood markers adjusted for age, gender and fat% on brain neurometabolites | | | | | |
| --- | --- | --- | --- | --- | --- |
|  | | LCModel | | Tarquin | |
|  |  | β | p-value | β | p-value |
| Log IL-6 o.e. | tNAA/tCr DPCC | -0.140 | 0.327 | -0.185 | 0.183 |
|  | tCho/tCr DPCC | 0.044 | 0.741 | -0.117 | 0.417 |
|  | Glx/tCr DPCC | 0.085 | 0.556 | 0.281 | 0.053 |
|  | mIns/tCr DPCC | 0.002 | 0.989 | 0.196 | 0.174 |
|  | tNAA/mIns DPCC | 0.032 | 0.829 | -0.268 | 0.057 |
|  | tNAA/tCr l HPC | 0.022 | 0.887 | -0.169 | 0.272 |
|  | tCho/tCr l HPC | -0.067 | 0.653 | -0.048 | 0.761 |
|  | Glx/tCr l HPC | 0.120 | 0.395 | 0.317 | 0.033* |
|  | mIns/tCr l HPC | -0.061 | 0.692 | 0.146 | 0.358 |
|  | tNAA/mIns l HPC | 0.069 | 0.651 | -0.287 | 0.067 |
|  | tNAA/tCr l SM1 | 0.245 | 0.074 | 0.103 | 0.465 |
|  | tCho/tCr l SM1 | 0.004 | 0.979 | -0.053 | 0.719 |
|  | Glx/tCr l SM1 | 0.086 | 0.554 | 0.067 | 0.650 |
|  | mIns/tCr l SM1 | 0.052 | 0.698 | 0.020 | 0.885 |
|  | tNAA/mIns l SM1 | 0.084 | 0.550 | 0.015 | 0.915 |
|  | tNAA/tCr l MTC | -0.407 | 0.011* | 0.066 | 0.126 |
|  | tCho/tCr l MTC | 0.442 | 0.007* | 0.161 | 0.322 |
|  | Glx/tCr l MTC | -0.147 | 0.366 | 0.425 | 0.006* |
|  | mIns/tCr l MTC | -0.090 | 0.563 | 0.048 | 0.764 |
|  | tNAA/mIns l MTC | -0.025 | 0.875 | -0.038 | 0.812 |
|  | tNAA/tCr r DLPFC | 0.085 | 0.524 | -0.056 | 0.694 |
|  | tCho/tCr r DLPFC | -0.065 | 0.644 | -0.061 | 0.675 |
|  | Glx/tCr r DLPFC | 0.249 | 0.083 | 0.292 | 0.052 |
|  | mIns/tCr r DLPFC | 0.157 | 0.247 | 0.097 | 0.504 |
|  | tNAA/mIns r DLPFC | -0.113 | 0.404 | -0.117 | 0.413 |
|  | tNAA DPCC | -0.063 | 0.649 | -0.118 | 0.404 |
|  | tCho DPCC | 0.057 | 0.698 | -0.070 | 0.643 |
|  | Glx DPCC | 0.108 | 0.455 | 0.266 | 0.062 |
|  | mIns DPCC | 0.030 | 0.838 | 0.180 | 0.205 |
|  | tCr DPCC | 0.037 | 0.777 | 0.071 | 0.623 |
|  | tNAA l HPC | -0.068 | 0.637 | -0.125 | 0.397 |
|  | tCho l HPC | -0.150 | 0.321 | -0.003 | 0.983 |
|  | Glx l HPC | 0.066 | 0.652 | 0.306 | 0.043* |
|  | mIns l HPC | -0.091 | 0.552 | 0.210 | 0.173 |
|  | tCr l HPC | -0.067 | 0.649 | 0.050 | 0.728 |
|  | tNAA l SM1 | 0.403 | 0.002* | 0.181 | 0.203 |
|  | tCho l SM1 | 0.100 | 0.488 | 0.080 | 0.582 |
|  | Glx l SM1 | 0.151 | 0.290 | 0.067 | 0.643 |
|  | mIns l SM1 | 0.136 | 0.290 | 0.135 | 0.307 |
|  | tCr l SM1 | 0.215 | 0.125 | 0.105 | 0.467 |
|  | tNAA l MTC | -0.486 | 0.002* | -0.258 | 0.102 |
|  | tCho l MTC | 0.289 | 0.070 | 0.157 | 0.329 |
|  | Glx l MTC | -0.221 | 0.194 | 0.433 | 0.005* |
|  | mIns l MTC | -0.171 | 0.304 | 0.068 | 0.668 |
|  | tCr l MTC | -0.132 | 0.402 | 0.019 | 0.905 |
|  | tNAA r DLPFC | 0.162 | 0.254 | 0.053 | 0.702 |
|  | tCho r DLPFC | -0.017 | 0.903 | 0.012 | 0.935 |
|  | Glx r DLPFC | 0.232 | 0.113 | 0.339 | 0.022* |
|  | mIns r DLPFC | 0.115 | 0.395 | 0.161 | 0.269 |
|  | tCr r DLPFC | 0.095 | 0.519 | 0.145 | 0.339 |
| * p < 0.05; Multiple linear regression analysis was used adjusted for age, gender and fat%. Abbreviations: DLPFC, dorsolateral prefrontal cortex; DPCC, dorsal posterior cingulate cortex; Glx, glutamate-glutamine complex; GMV, gray matter volume; HPC, hippocampal cortex; IL-6, interleukin-6; l, left; mIns, myoinositol; MTC, medial temporal cortex; o.e., outlier excluded; r, right; SM1, primary sensorimotor cortex; tCho, total choline; tCr, total creatine, tNAA, total N-acetyl aspartate | | | | | |

| **Supplementary table 10.** The effect of inflammatory blood markers adjusted for age, gender and fat% on brain neurometabolites | | | | | |
| --- | --- | --- | --- | --- | --- |
|  | | LCModel | | Tarquin | |
|  |  | β | p-value | β | p-value |
| Kynurenine | tNAA/tCr DPCC | -0.060 | 0.646 | -0.031 | 0.813 |
|  | tCho/tCr DPCC | 0.148 | 0.237 | 0.069 | 0.596 |
|  | Glx/tCr DPCC | -0.033 | 0.804 | 0.028 | 0.833 |
|  | mIns/tCr DPCC | 0.013 | 0.926 | 0.178 | 0.188 |
|  | tNAA/mIns DPCC | -0.005 | 0.972 | -0.169 | 0.201 |
|  | tNAA/tCr l HPC | -0.054 | 0.705 | -0.043 | 0.767 |
|  | tCho/tCr l HPC | 0.121 | 0.368 | 0.048 | 0.746 |
|  | Glx/tCr l HPC | 0.206 | 0.119 | 0.165 | 0.261 |
|  | mIns/tCr l HPC | 0.150 | 0.285 | -0.023 | 0.877 |
|  | tNAA/mIns l HPC | -0.203 | 0.145 | -0.097 | 0.519 |
|  | tNAA/tCr l SM1 | -0.133 | 0.288 | -0.107 | 0.411 |
|  | tCho/tCr l SM1 | 0.218 | 0.103 | 0.063 | 0.640 |
|  | Glx/tCr l SM1 | -0.066 | 0.626 | 0.022 | 0.871 |
|  | mIns/tCr l SM1 | 0.193 | 0.128 | 0.317 | 0.012* |
|  | tNAA/mIns l SM1 | -0.199 | 0.119 | -0.255 | 0.039* |
|  | tNAA/tCr l MTC | -0.312 | 0.026* | -0.242 | 0.078 |
|  | tCho/tCr l MTC | 0.418 | 0.003* | 0.254 | 0.072 |
|  | Glx/tCr l MTC | 0.224 | 0.119 | 0.172 | 0.222 |
|  | mIns/tCr l MTC | -0.022 | 0.870 | -0.044 | 0.747 |
|  | tNAA/mIns l MTC | -0.081 | 0.556 | -0.115 | 0.407 |
|  | tNAA/tCr r DLPFC | -0.106 | 0.387 | -0.159 | 0.229 |
|  | tCho/tCr r DLPFC | 0.297 | 0.020* | 0.243 | 0.063 |
|  | Glx/tCr r DLPFC | 0.107 | 0.427 | -0.052 | 0.717 |
|  | mIns/tCr r DLPFC | 0.214 | 0.095 | 0.108 | 0.427 |
|  | tNAA/mIns r DLPFC | -0.235 | 0.056 | -0.172 | 0.190 |
|  | tNAA DPCC | -0.030 | 0.812 | 0.147 | 0.249 |
|  | tCho DPCC | 0.143 | 0.286 | 0.188 | 0.162 |
|  | Glx DPCC | 0.003 | 0.982 | 0.119 | 0.369 |
|  | mIns DPCC | 0.026 | 0.849 | 0.314 | 0.016* |
|  | tCr DPCC | 0.019 | 0.877 | 0.226 | 0.080 |
|  | tNAA l HPC | -0.239 | 0.076 | -0.317 | 0.022* |
|  | tCho l HPC | -0.133 | 0.336 | -0.283 | 0.052 |
|  | Glx l HPC | 0.018 | 0.896 | 0.088 | 0.556 |
|  | mIns l HPC | -0.047 | 0.738 | -0.209 | 0.155 |
|  | tCr l HPC | -0.183 | 0.175 | -0.379 | 0.005* |
|  | tNAA l SM1 | 0.015 | 0.903 | -0.017 | 0.899 |
|  | tCho l SM1 | 0.319 | 0.015* | 0.169 | 0.204 |
|  | Glx l SM1 | -0.003 | 0.984 | 0.037 | 0.787 |
|  | mIns l SM1 | 0.278 | 0.020* | 0.375 | 0.002* |
|  | tCr l SM1 | 0.204 | 0.116 | 0.145 | 0.281 |
|  | tNAA l MTC | -0.364 | 0.007* | -0.270 | 0.053 |
|  | tCho l MTC | 0.275 | 0.049* | 0.195 | 0.165 |
|  | Glx l MTC | 0.130 | 0.383 | 0.233 | 0.108 |
|  | mIns l MTC | -0.093 | 0.518 | -0.003 | 0.983 |
|  | tCr l MTC | -0.138 | 0.315 | -0.018 | 0.898 |
|  | tNAA r DLPFC | 0.071 | 0.592 | 0.024 | 0.853 |
|  | tCho r DLPFC | 0.436 | 0.001* | 0.386 | 0.002* |
|  | Glx r DLPFC | 0.164 | 0.222 | 0.015 | 0.917 |
|  | mIns r DLPFC | 0.302 | 0.014* | 0.250 | 0.063 |
|  | tCr r DLPFC | 0.194 | 0.145 | 0.244 | 0.075 |
| * p < 0.05; Multiple linear regression analysis was used adjusted for age, gender and fat%. Abbreviations: DLPFC, dorsolateral prefrontal cortex; DPCC, dorsal posterior cingulate cortex; Glx, glutamate-glutamine complex; GMV, gray matter volume; HPC, hippocampal cortex; l, left; mIns, myoinositol; MTC, medial temporal cortex; o.e., outlier excluded; r, right; SM1, primary sensorimotor cortex; tCho, total choline; tCr, total creatine, tNAA, total N-acetyl aspartate | | | | | |
